# Facilitating safe transition to home for preterm infants (FAST home): Protocol for a retrospective observational study

**DOI:** 10.1371/journal.pone.0318309

**Published:** 2025-02-11

**Authors:** Lisa Szatkowski, Janine Abramson, Sha Tao, Sarah E. Seaton, Jon Dorling, Michelle Arellano-Meza, Jane Harvey, Tom Harvey, Shalini Ojha

**Affiliations:** 1 Centre for Perinatal Research, School of Medicine, University of Nottingham, Nottingham, United Kingdom; 2 Department of Population Health Sciences, University of Leicester, Leicester, United Kingdom; 3 Neonatal Unit, Southampton General Hospital, University Hospital Southampton NHS Trust, Southampton, United Kingdom; 4 Parent Collaborator, United Kingdom,; 5 Neonatal Unit, University Hospitals of Derby and Burton NHS Trust, Derby, United Kingdom; PLOS: Public Library of Science, UNITED KINGDOM OF GREAT BRITAIN AND NORTHERN IRELAND

## Abstract

**Background:**

Preterm infants (i.e., those born before 37 completed weeks of pregnancy) often require additional care and are admitted to neonatal units soon after birth. Readiness for discharge home typically requires a level of physiological maturity such that an infant is able to: 1) breathe spontaneously without additional support; 2) maintain their own body temperature; 3) take all their nutritional requirements orally; 4) weighs ≥ 1700g and is gaining weight. Longer hospital stays than necessary can be detrimental to infants, stressful for families, and costly. Currently, little is known about whether, how long and why preterm infants stay in hospital beyond physiological readiness for discharge.

**Materials and methods:**

We will conduct a retrospective cohort study using data from the National Neonatal Research Database on all infants born at < 37 weeks’ gestational age (GA) admitted to neonatal units in England and Wales from 2016-2022. The day of life and postmenstrual age infants reach each physiological milestone, and the final barrier to discharge, will be identified. We will assess whether the final barrier differs by GA and between neonatal Operational Delivery Networks and summarise the number of days infants remain in hospital after surpassing all physiological milestones. We will explore the characteristics of infants, mothers and neonatal units associated with extended hospital stays beyond physiological readiness for discharge.

**Discussion:**

The results of this study will allow identification of areas to target to help achieve a safe reduction in length of hospital stay and will support the development of evidence-based recommendations to guide optimal discharge practices.

**Trial registration:**

ClinicalTrials.gov NCT06284044

## Introduction

Infants born preterm (before 37 completed weeks of pregnancy) often require additional care and may be admitted to a neonatal unit soon after birth. Depending on their gestational age (GA) at birth and medical needs, preterm infants have varying lengths of hospital stay (LOS) from days to months. Most go home between 37 and 40 weeks’ post-menstrual age (PMA, the time elapsed between the first day of the mother’s last menstrual period and the infant’s current age) [1].

Although neonatal care is life saving and the survival of infants born very preterm has improved markedly over time [2], staying in hospital longer than necessary can be detrimental. Bright lights, noise, and lack of one-to-one care can adversely impact infant development [3]. Prolonged hospitalisation means prolonged family separation and continued parental stress [4]. Having a child in hospital incurs unanticipated and unbudgeted costs. Bliss, the UK’s largest charity representing families of sick and preterm infants, estimated this to be £2,256 for the average 8 weeks of neonatal stay in 2008 [5]. With cost-of-living increases, this is likely to be significantly higher now. Prolonged hospitalisation is also costly to the health service. In the UK, one day of neonatal ‘special care’ costs from £727.72 (with a carer, e.g., a parent present to support nursing staff) to £1222.84 (without an external carer present) [6]. Around 39,000 preterm infants are admitted to UK neonatal units each year [7]. If LOS could be reduced by just one day per infant, the NHS would save a minimum of £28 million per year.

Reducing LOS would also help alleviate the constant pressure in UK neonatal units for cot space and staff [5]. In 2014/15 (the most recent available data), 15% of all emergency transfers between neonatal units (974 infants) were due to lack of capacity at the transferring neonatal unit rather than due to medical need [5]. On one weekday in 2019, 10% of neonatal units had gaps in medical staffing and 15% gaps in nurse staffing [8]. These pressures expose babies to unnecessary risk from inadequate staff-patient ratios and harms from transfer. Preterm infants transferred in the first few days after birth have increased risk of brain injury [9] and transfers worsen anxiety and economic hardship for families who travel long distances to be with their infants [5].

There are no formal UK recommendations for criteria for discharge of preterm infants. Whilst there are some recommendations from similar settings (e.g., Canada [10]) there is no accepted consensus. However, ‘discharge readiness’ typically requires a level of physiological maturity such that the infant can:

1. Breathe on their own: preterm infants often have pauses in their breathing (apnoea of prematurity) and many require breathing support. As they mature, infants can breathe on their own. Some may continue to need additional oxygen, and some go home on oxygen therapy.
2. Maintain body temperature: initially preterm infants are nursed in incubators. As their ability to generate and preserve heat matures, they move into heated cots and finally into regular cots with normal clothing and blankets.
3. Feed adequately: preterm infants take time to establish feeding. Initially they may need parenteral nutrition. Milk is slowly introduced via a gastric tube. As their ability to suck and swallow matures, they learn to feed orally (on the breast or by bottle). To be discharged without additional support, infants should be able to take their milk requirements orally. Occasionally, where the service is available, infants can go home with partial nasogastric feeding.
4. Gain weight: most infants are considered ready for discharge if they consistently gaining weight and are above a certain weight cut-off, typically 1700-1800g.

Infants mature at variable pace and may become mature in some aspects but not all, e.g., be able to breathe well, maintain their temperature, and gain weight, but still be unable to take sufficient oral feeds. The final reason that keeps an infant in hospital is referred to as the ‘final discharge barrier’ [11]. Apart from our recent single-centre audit [12], little is known about when infants reach each of the physiological milestones listed above, or what is the most common final discharge barrier for preterm infants in the UK. Knowing what the most frequent barriers are that keep infants in hospital longer will help inform and develop targeted interventions that are most likely to support safe and early discharge home.

Studies from elsewhere have highlighted variations in LOS and the potential to intervene with strategies to support safe, early discharge. In California, infants born at 30-34 weeks’ GA are discharged, on average, 4 days earlier compared to the UK, a difference attributed to the integrated healthcare approach implemented by the Kaiser Permanente Medical Group, which requires an understanding of the reasons discharge is delayed [13]. In another study in the USA, independent oral feeding was identified as the final discharge barrier and a Quality Improvement (QI) project to support independent oral feeding reduced the average LOS by 1 week [11].

Earlier discharge can be safe, improve infant and family well-being [4,14] and simultaneously save healthcare resources. The FAST Home Study will analyse routinely recorded clinical data held in electronic patient records (EPR) to investigate LOS for preterm infants and identify the final discharge barrier(s) that require preterm infants to remain in hospital the longest. We will explore the characteristics of infants, mothers and neonatal units that are associated with extended hospital stays beyond physiological readiness for discharge. The results will help us identify and target areas to focus work to enable sooner and safer discharge.

### Objectives

#### Primary objectives.

1. Identify the final discharge barrier that requires preterm infants to remain in hospital the longest.
2. Identify the age and PMA when preterm infants receiving neonatal care reach each of the physiological criteria for discharge.

#### Secondary objectives.

1. Investigate the difference between the time when each infant was physiologically ready for discharge and the time of actual discharge.
2. Investigate the characteristics of infants, mothers and care providers associated with length of hospital stay and prolonged stay after reaching physiological readiness for discharge.

## Materials and methods

### Design

Retrospective cohort study of routinely collected data.

### Setting

Neonatal units in England and Wales.

### Time period

Babies born from 01 January 2016 to 31 December 2022.

### Data source

Routinely recorded EPR data from all admissions to NHS neonatal units in England and Wales, held within the National Neonatal Research Database (NNRD). Data are entered by clinical staff at the point of care and extracted, anonymised and combined by the Neonatal Data Analysis Unit (NDAU) at Imperial College, London.

### Eligibility criteria

We will include infants who were: born at < 37 weeks GA between 01 January 2016 and 31 December 2022; admitted for neonatal care for least 48 hours; discharged home alive. We will exclude infants: with missing data on key characteristics (e.g., sex, gestational age, final discharge destination) or missing 1 or more episodes of care; born with a lethal congenital anomaly (defined by clinical expertise and existing literature [15]); admitted late to neonatal care (>24 hours after birth for infants < 34 weeks GA and > 7 days for infants 34–36 weeks GA).

### Intervention

None – investigation of routine care.

### Primary outcomes

We will report outcomes overall and by GA group (22–24 weeks – most preterm; 25–27 weeks – extremely preterm; 28–31 weeks – very preterm; 32–33 weeks – moderately preterm; 34–36 weeks – late preterm), single completed GA week, and neonatal Operational Delivery Network [16] (ODN).

1. Final discharge barrier, i.e., the last of the physiological barriers listed below to be reached.
2. Chronological age (in days since birth) and PMA, when infants:
  a. Were breathing spontaneously without any additional oxygen support and without having received caffeine (used to treat apnoea of prematurity) for at least 5 days;
  b. Achieved full oral feeding without need for intravenous or gastric feeding;
  c. Achieved a weight of ≥ 1700 grams.

These data items are almost fully recorded daily during routine clinical care as they are used either to guide care (e.g., in the calculation of weight-dependent nutritional requirements and drug dosages) or are used to categorise the intensity of care and thereby used to determine neonatal unit funding. Data on when an infant is able to independently maintain their body temperature is not routinely recorded in EPRs and hence are not included. A recent audit at the neonatal unit in the Royal Derby Hospital showed that, over a 4-month period, no infant had their discharge delayed due to need for additional temperature support.

### Secondary outcomes

1. Number of days infants remain in hospital after surpassing all physiological discharge barriers.
2. Association between length of hospital stay and number of days of care after surpassing all physiological discharge barriers and:
  a. Infant characteristics, including sex, GA, condition at birth, co-morbidities;
  b. Maternal characteristics, including age, ethnicity and Index of Multiple Deprivation quintile;
  c. Neonatal unit characteristics, including unit level and admissions per year.
3. For infants who remain in hospital after surpassing all physiological discharge barriers we will explore reason(s) for prolonged stay, e.g., continued IV medication, surgical procedures, social care concerns.

### Sample size calculation

We will use all available data. Approximately 34,000 preterm infants are admitted to neonatal units in England and Wales per year. Over our 7-year study period this equates to ~ 238,000 infants, including ~ 4,660 of the most preterm infants born at 22–24 weeks’ GA and ~ 11,830 infants born at 25–27 weeks’ GA. Even in these smallest sub-groups we expect to be able to estimate the proportion of infants with a particular discharge barrier with a margin of error of < 1%.

### Statistical analysis

#### Description of study population.

After data cleaning, exclusions, overall and for each individual exclusion criterion (see eligibility criteria), will be tabulated and the final study population size for analysis determined. Descriptive statistics will be presented to summarise the demographic and clinical characteristics of the study population, overall and by GA group (22–24 weeks – most preterm; 25-27 weeks – extremely preterm; 28–31 weeks – very preterm; 32–33 weeks – moderately preterm; 34–36 weeks – late preterm) and neonatal ODN [16].

#### Analysis of primary outcomes.

The day of life and PMA infants achieve each of the three physiological criteria for discharge for which data are routinely recorded will be identified and summarised as appropriate (mean ±  SD or median ±  IQR), overall and by GA group, single completed GA week and ODN. For each infant we will identify the final barrier to discharge as the last of the milestones to be achieved. If an infant reached two or more milestones on the same day, all the relevant events will be noted as the final barrier. We will quantify the number and percentage of infants who had each of the 3 aspects identified as their final discharge barrier, overall and by GA group, single week of GA and ODN, and assess whether the final discharge barrier differs between groups. Some infants are discharged prior to surpassing all physiological milestones, e.g., an infant may be < 1700 g or go home on nasal canula oxygen or with partial nasogastric feeding. The frequency of such discharges will be reported and variations between groups assessed. Some infants, particularly those born at later gestations, may have met all physiological criteria at birth but be admitted to neonatal care for other reasons; again, we will assess the frequency of this occurrence.

We will explore the use of descriptive approaches, such as sequence analysis [17] and latent class analysis [18], to summarise and describe the trajectory(ies) which infants follow in reaching each physiological milestone, their final discharge barrier and are then discharged home.

#### Analysis of secondary outcomes.

For each infant we will count the number of days they remain in hospital after surpassing all physiological discharge barriers and summarise this using appropriate descriptive statistics, overall, by GA group, single completed GA week and by ODN. For infants who are discharged home weighing < 1700g, being fed via nasogastric tube or with home oxygen, we will count and summarise the number of days the infant remains in hospital after surpassing all but this milestone. For infants who stay in hospital after surpassing all physiological discharge barriers we will use all available data recorded in the NNRD to explore and summarise what happens to them during this period, to help identify any reason(s) for the prolonged stay, such as: pre-existing and new diagnoses; medication use, such as continued administration of IV medications; surgical procedures and other cot-side interventions; ongoing care for major congenital anomalies; social factors, such as maternal drug/alcohol misuse or social care involvement. We will use regression techniques to explore the association between infant, maternal and unit characteristics (selected a priori based on clinical knowledge and existing literature) and length of hospital stay and length of stay after surpassing all physiological milestones.

#### Missing data.

We will report the number of infants with missing data for each of the three physiological criteria for discharge and for whom it is not possible to determine the final discharge barrier or the number of additional days they remain in hospital. As described above, we expect the amount of missing outcome data to be small as these data items are almost fully recorded daily during routine clinical care. We will describe the amount of missing data and assess the missingness mechanism for infant, maternal and unit-level covariates describing the study population. For the regression analysis we will use an appropriate technique to account for missing data depending upon the amount missing and reason, such as treating missing values as a separate category or multiple imputation.

### Ethical approval

The study is sponsored by the University Hospitals of Derby and Burton NHS Foundation Trust and has been registered on the ClinicalTrials.gov database (Ref: NCT06284044). The study has been approved by the South East Scotland Research Ethics Committee 01 and the Health Research Authority and Health and Care Research Wales (IRAS 323099). Parents are offered the opportunity to opt out of their child’s data being held in the NNRD, and every neonatal unit contributing to the NNRD is informed about all new studies and asked if they wish to be included.

### Study status and timeline

The study does not involve direct recruitment of participants or record screening. Raw NNRD data have already been received from NDAU; data cleaning and extraction of variables needed for analysis has commenced and is expected to be complete by mid-2025. Results are expected by summer 2025.

### Patient, professional and public involvement

This study originated from PPI consultations carried out during the set-up of the FEED1 trial [19], a large, ongoing randomised controlled trial of early enteral feeding for preterm infants. In a survey of 248 parents, we found that length of hospital stay was overwhelmingly the major focus for families. Over 80% said that research that could help get infants home sooner should be a priority. They said that reducing the time spent in the hospital “by even a day” would improve parental and family wellbeing significantly. Our ongoing PPI work with parent groups such as Bump2Baby and Derby Bliss groups further supports this.

We initially planned to include only infants born at < 34 weeks’ GA as they are routinely admitted for neonatal care. However, on consultation, parents said all preterm infants should be included. They felt that although not all infants born at 34–36 weeks’ GA need neonatal care, those who do have similar problems to more immature infants. Therefore, study inclusion criteria were changed to include all infants born at < 37 weeks’ GA who are admitted for neonatal care.

Our study team, parent advisory group and authors of this protocol include the parents of preterm infants who received neonatal care. Their perspective, in their own words, is summarised in Table 1.

**Table 1:** Parent co-investigators’ perspectives on lived experience of neonatal care.

Parent 1
While in the unit, gaining weight was one of the biggest concerns. Our baby was not gaining weight at the expected rate, and was placed on a strict three-hour feeding schedule. This particular situation eroded my confidence in my abilities to feed my baby and the healthcare system in general. While breastfeeding was encouraged, it was not supported adequately. We needed clear and candid communication from the healthcare staff—nurses, doctors, and specialists—because uncertainty only heightened our stress and anxiety, leading to mistrust and frustration with the system. When concerns are downplayed or not properly explained, it paints an incomplete picture for parents, potentially prolonging hospital stays or leading to readmissions.
**Parents 2**
Our second son was born by emergency c section, due to pre-eclampsia. He was intubated at birth. We understood that we would stay on the NICU for a few weeks. I was expressing milk to feed him through an NG tube, in addition to breast feeding him. I also still had high blood pressure and was suffering migraine like headaches due to constant changes in medication. Our elder son was still at home being looked after by grandparents. We could not go home until our new baby had gained sufficient weight and was having at least half his feeds orally. He also struggled to maintain his temperature, partly because the ward was so cold due to the old buildings. It was two weeks before we were able to come home, to finally be a family of 4 in our own home. We would have loved to have a better understanding of when we might go home safely. Once we got home the transition was difficult. We had repeated readmissions and our GP said she had never treated a baby so small. It took us at least a few months before we felt safe, and life got back to normal again.

## Discussion

The results of this study will allow ODNs to identify local priorities and areas to target to achieve a safe reduction in length of hospital stay, and at a national level will support the development of evidence-based recommendations to guide optimal discharge practices in all neonatal units. The results will identify the discharge barrier that is the reason most infants stay in hospital longer and inform the development of interventions that could help infants and their families overcome these barriers sooner and therefore go home early.

These benefits will directly improve the lives of preterm infants and their families by decreasing unnecessary separation and empowering parents to take their babies home sooner. Reduced hospital stay will improve cot capacity, reduce transfers for non-medical reasons, and families will avoid the anxiety and cost associated with having to travel long distances. Neonatal services will benefit from improved patient flow and more productive use of resources such as nursing time and transport teams. The NHS will benefit from significant cost savings from the fewer days of care and avoided transfers. The benefits of reduced burden on families, and improved care of preterm infants, will improve long term outcomes that have benefits for the wider society.

The results will inform future work to understand the interplay between physiological readiness for discharge and family readiness to care for their child independently (including keeping the baby warm, feeding, bathing, safe travel, and giving medicines) and being emotionally ready and confident to take their infant home. This will support the development of discharge guidelines that reunite families at home as soon as is safely possible, balanced against the risk of unintended consequences such as emergency hospital readmission.

### Dissemination plans

We will use the RECORD checklist [20], an extension of the STROBE guidelines [21], to ensure transparency of reporting of our methods and results. On completion of the study, findings will be disseminated via: a detailed report for the NIHR as well as an individually tailored written report for each of the 11 neonatal ODNs in England and Wales; public facing materials (short animated video, infographic) made available in common UK languages, created with and shared via PPI partners; open access, peer-reviewed journals and scientific conferences; and dissemination to relevant specialised groups including the British Association of Perinatal Medicine (BAPM), the Neonatal Specialised Commissioning Group and the Maternity and Neonatal Safety Improvement Programme.
